# High Levels of Perfluorooctane Sulfonate in Children at the Onset of Diabetes

**DOI:** 10.1155/2015/234358

**Published:** 2015-05-05

**Authors:** Barbara Predieri, Lorenzo Iughetti, Cristiana Guerranti, Patrizia Bruzzi, Guido Perra, Silvano E. Focardi

**Affiliations:** ^1^Department of Medical and Surgical Sciences of the Mother, Children and Adults, University of Modena and Reggio Emilia, 41124 Modena, Italy; ^2^Department of Environmental Sciences “G. Sarfatti”, University of Siena, 53100 Siena, Italy

## Abstract

*Background*. Impairments of endocrine system may be associated with exposure to perfluorinated compounds that are able to bind nuclear receptors, including the peroxisome proliferator-activating receptors. Aim of this study was to assess perfluorooctane sulfonate and perfluorooctanoic acid concentrations in children and adolescents at the onset of type 1 diabetes compared to healthy controls. *Methods*. Forty-four children and adolescents were recruited and subdivided into two groups: (A) 25 subjects with type 1 diabetes and (B) 19 healthy controls. Perfluorinated compounds were measured using high performance liquid chromatography with electrospray ionization tandem mass spectrometry. Nonparametric statistical analysis was performed. *Results*. Perfluorooctane sulfonate concentrations were significantly higher in patients with type 1 diabetes compared to controls (1.53 ± 1.50 versus 0.55 ± 0.15 ng/mL, resp.; *p* < 0.001). Multivariate linear regression analysis identified lipid levels as significant predictive factors for perfluorooctane sulfonate levels. *Conclusions*. Our data suggests that higher serum levels of perfluorooctane sulfonate may be considered a biomarker of exposure and susceptibility to develop type 1 diabetes.

## 1. Introduction

Perfluorinated compounds (PFCs) are chemical products extensively investigated for their environmental ubiquity and toxicity. Two of the PFCs of most concern are the eight-carbon-chain perfluorooctane sulfonate (PFOS) and perfluorooctanoic acid (PFOA), which are synthetically produced or derived by the metabolism of other PFCs and are widespread since used in industrial and consumer products [1]. PFOS and PFOA are both lipo- and hydrophobic and are characterized by a high potential to bioaccumulate after absorption, binding serum proteins rather than storing in lipids [2]. They are slowly metabolized and their half-life in human blood serum has been estimated to be more than 5 years for PFOS and around 4 years for PFOA [3]. This extremely long half-life in humans contrasts with the relatively rapid elimination seen in animal models drawing attention to potential risks for human health [2].

PFCs are globally found in human tissues, as humans are daily exposed to contaminated food, water, and air, independently to industries nearby [4, 5]. Human biomonitoring of the general population in different countries has shown that PFOS and PFOA may also be found in breast milk, liver, seminal fluid, and umbilical cord blood [5–7]. However, in spite of the widespread exposure to PFCs, there are considerable individual differences in exposure levels [8].

The effects of exposure to PFOS and/or PFOA on human health have not been fully ascertained yet, but by extrapolating animal data they seem to be related to pathological conditions in exposed organisms including enlargement of the liver, dyslipidemia, neurobehavioral toxicity, immune system toxicity, reduced body weight, reproductive toxicity, and hormonal effects [5]. Specifically, alterations in hepatic metabolism and function have been attributed to the ability of PFOS and PFOA to bind nuclear receptors, including the peroxisome proliferator-activating receptor-*α* (PPAR*α*) [9] and to disrupt serum protein ligand binding [10], acting as potential endocrine disruptors [11] although it is still unclear whether these animal-based evidences can be extrapolated to humans.

Compared with effects seen in animals, human studies have reported different associations between PFCs and both lipid levels [12, 13] and the endocrine system, especially thyroid function [12]. Olsen and Zobel [12] measured PFOA concentrations in 506 male fluorochemical production workers demonstrating no association with total cholesterol (TC), low-density lipoprotein cholesterol (LDL), hepatic enzymes, or thyroid hormones. Another study including 12476 children and adolescents showed that PFOA was significantly associated with increased TC and LDL, and PFOS was significantly associated with increased TC, high-density lipoprotein cholesterol (HDL), and LDL [13].

Both prenatal and childhood elevated exposure to PFCs were linked to reduced humoral immune response to routine immunizations in early childhood, suggesting an immune system alteration [14, 15]. Children with high blood levels of PFCs had lower antibody levels for diphtheria and tetanus than children with lower PFC levels. The antibody loads were likely too low to protect children against these infections [14].

Up to now no study exists investigating the potential relationship between PFCs compounds exposure and development of an endocrine-autoimmune disease in childhood and adolescents. For this purpose, we studied a group of children and adolescents at the onset of type 1 diabetes (T1DM) compared to healthy controls who were consecutively recruited during 2 years (2012-2013).

## 2. Methods

### 2.1. Study Population

We performed a case-control study at the Pediatric Department of the University of Modena and Reggio Emilia (Italy). Study subjects were 25 children and adolescents enrolled at the onset of T1DM (3.15–13.1 years, 12 males; median glycohemoglobin 10.5%) and 19 healthy subjects used as control group (1.88–13.6 years, 9 males). Each subject included in the control group was referred to our attention because of short stature; after appropriate investigations, endocrine or other diseases were excluded. To avoid confounding factors due to different PFCs exposure each control was recruited at the same time (or +2 weeks) of each T1DM onset. All subjects were native Italians and residents in Modena or surrounding areas at least 5 years at the time of their recruitment in the study. Participation and enrollment included collection of auxological data together with blood samples. Written informed consent was obtained from all parents at the moment of recruitment in the study and before data collection. The design of the study was approved by the Ethics Committee of the University of Modena and Reggio Emilia (Protocol number 1429CE).

### 2.2. Anthropometric Measurements

All patients underwent a complete clinical history and physical examination including anthropometric measurements that were performed by fully trained examiners according to the Anthropometric Standardization Reference Manual [16]. Height was measured to the nearest 0.1-cm with a calibrated wall-mounted stadiometer (Harpenden, Crymych, UK) and weight was measured to the nearest 0.1-kg with a calibrated scale. Body mass index (BMI) was calculated by dividing weight in kg by height squared (m^2^); *z*-score of BMI (*z*-BMI) was calculated using the appropriate Italian growth reference (ISPED Growth Calculator).

### 2.3. Laboratory Methods

Clinical laboratory tests were performed in each patient between 07.00 and 10.00 am, after a 12-hour overnight fast: leukocyte count (WBC), lymphocytes count, alanine aminotransferase (ALT), aspartate aminotransferase (AST), creatinine, urea, TC, HDL, LDL, triglycerides (TG), thyroid-stimulating hormone (TSH), PFOS, and PFOA.

Serum samples (about 0.5 mL) to assay PFCs were collected and stored few days after T1DM was diagnosed. Samples were kept frozen at −20°C until analysis; precautions were taken to avoid contamination. The analytical procedure for PFOS and PFOA follows Governini et al. [17]. Concentrations of PFCs were measured using high performance liquid chromatography (HPLC) with electrospray ionization (ESI) tandem mass spectrometry. Analyte separation was performed using a Finnigan Surveyor Plus HPLC System. Chromatographic separation was achieved using a Betasil© C18 column (Thermo Electron Corporation, San Jose, CA). For quantitative determination, the HPLC system was interfaced to a Finnigan LTQ linear ion trap mass spectrometer (Thermo 150 Electron Corporation, San Jose, CA) operated in negative electrospray mode. Instrumental parameters were optimized to transmit the [M-H]-ion for all the analytes. The repeatability and reproducibility were performed in triplicate and were 85% and 90%, respectively. The limit of detection for both PFOS and PFOA was 0.4 ng/mL. The laboratory staff was blinded to any information about the subjects.

### 2.4. Statistical Analysis

All results are reported as the mean ± standard deviation (SD). Data were checked for normal distribution using the Kolmogorov-Smirnov test, so nonparametric statistical analysis (STATISTICA software, StatSoft Inc., Tulsa, OK, USA) was performed. Between-group and gender comparisons were evaluated using the Mann-Whitney *U* test while between-variable differences (PFOS and PFOA) were analyzed through the Wilcoxon Matched Pairs Test. Spearman's correlation analysis was performed to assess the linear association between PFOS or PFOA and age, *z*-BMI, renal function, liver function, lipid profile, and TSH. The association between potential predictors and levels of PFOS or PFOA was evaluated using the following multivariate linear regression model including age, *z*-BMI, TC, HDL, TG, and LDL. Statistical significance was set at *p* < 0.05.

## 3. Results

The description of the study population is given in Table 1.

Mean TC levels were significantly higher in T1DM patients than in controls (183.7 ± 27.0 versus 170.1 ± 15.1 mg/dL, resp.; *p* = 0.015), with 24% of values classified as acceptable (<170 mg/dL). The lymphocyte count in T1DM was significantly lower than in controls (2.28 ± 0.60 versus 2.95 ± 0.85 migl/*μ*L, resp.; *p* = 0.008) with no apparent difference in WBC counts (Table 2). However, values were always within the normal range.

**Figure 1:**
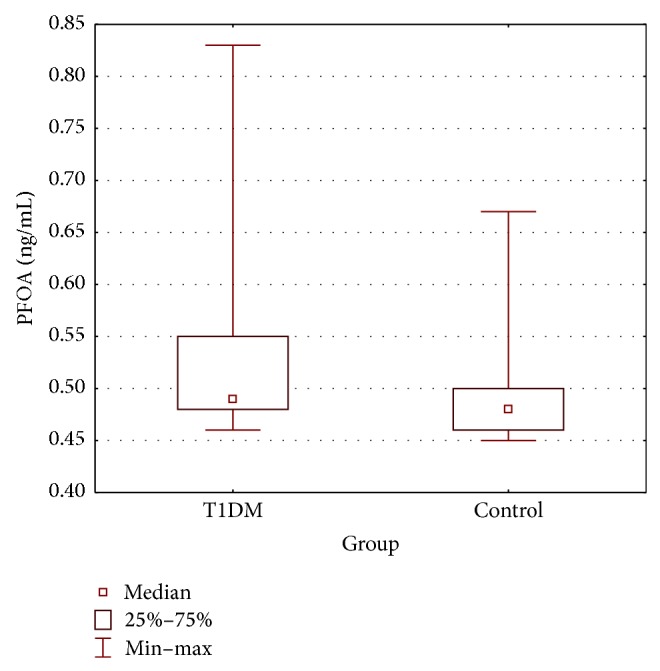
Serum concentration of PFOA (T1DM group compared with control group: Mann-Whitney *U* test *p* = 0.160).

**Figure 2:**
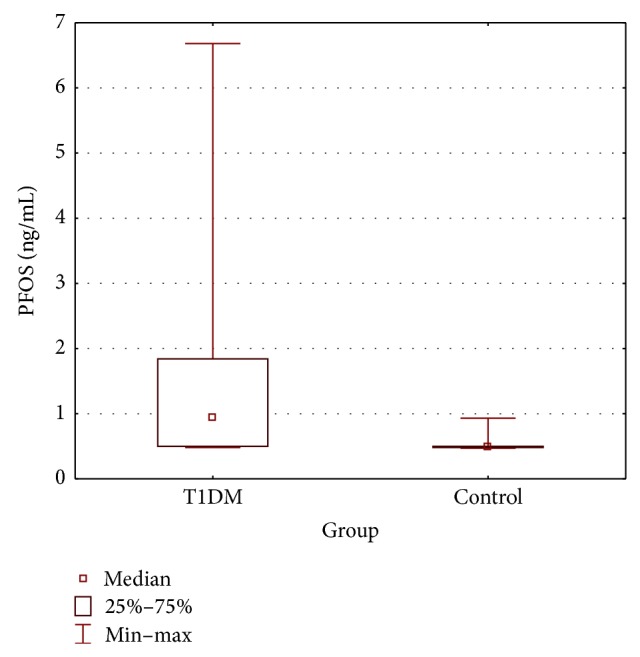
Serum concentration of PFOS (T1DM group compared with control group: Mann-Whitney *U* test *p* < 0.001).

PFOA concentrations were similar between T1DM and control groups (0.53 ± 0.09 versus 0.50 ± 0.06 ng/mL, resp.; *p* = 0.160) (Table 3 and Figure 1) while PFOS levels were significantly higher in T1DM patients (1.53 ± 1.50 versus 0.55 ± 0.15 ng/mL, resp.; *p* < 0.001) (Table 3 and Figure 2). All values of PFOA and PFOS were above the lower limit of detection (0.4 ng/mL) at that time. In T1DM patients PFOA concentrations ranged from 0.46 to 0.83 ng/mL with median values of 0.49 ng/mL, while PFOS concentrations ranged from 0.48 to 6.68 ng/mL with median values of 0.95 ng/mL. In the control group PFOA concentrations ranged from 0.45 to 0.67 ng/mL with median values of 0.48 ng/mL, while PFOS concentrations ranged from 0.47 to 0.93 ng/mL with median values of 0.49 ng/mL.

Using the Wilcoxon Matched Pairs Test we found that PFOA levels were significantly lower than PFOS ones in both T1DM (*p* < 0.001) and control (*p* = 0.048) groups.

In T1DM group, when data were analyzed according to gender, we did not find any difference in all analyzed variables, specifically PFCs (data not shown).

In T1DM group analyzing data using Spearman's correlation test we found a significant negative association between PFOS and TG levels (*r* = −0.50, *p* = 0.010) and ALT levels (*r* = −0.47, *p* = 0.016) while a significant positive association was demonstrated with creatinine values (*r* = 0.49, *p* = 0.012). PFOA levels were positively correlated with AST (*r* = 0.42, *p* = 0.036). Data for controls demonstrated a significant positive association between PFOS levels and age (*r* = 0.50, *p* = 0.035) and TSH (*r* = 0.57, *p* = 0.012).

Finally, considering the whole study population, multivariate linear regression analysis allowed us to identify TC (*β* = 0.82, *p* = 0.040) and TG (*β* = −0.44, *p* = 0.029) as predictive factors for PFOS levels; none of included variables were demonstrated to be significant predictive factors for PFOA concentrations. Age was shown to be the only significant predictive factor for PFOS levels in T1DM subjects (*β* = 0.43, *p* = 0.036), while, in the control group, regression analysis did not show statistically significance for any predictive factor. No significant predictive factors were demonstrated for PFOA neither in T1DM nor in control groups (Table 4).

## 4. Discussion

To the best of our knowledge, no study has yet investigated the potential relationship between PFCs exposure and development of autoimmune diseases in children and adolescents. This is the first study about the presence of serum PFCs in patients at T1DM onset compared to healthy controls.

We found that mean serum PFOS concentrations were significantly higher in T1DM subjects than in controls. Moreover, PFOA levels were significantly lower than PFOS ones. PFOA levels have been generally measured as slightly lower than PFOS, with relevant differences in terms of frequency among several studies, probably related to the huge variability of detection methods. In the PERFORCE study [18], 17 participating laboratories produced standardized serum cutoffs that varied with a relative SD of 31.5%. One strength of our study is the high sensitivity of the equipment we used, which allowed us to determine PFOA concentrations in all the analyzed serum samples, even at extremely low concentrations.

Despite significantly lower PFOS levels our controls showed a significant positive correlation between PFOS concentrations and chronological age; the same was not found in patients with T1DM. The meaning of this correlation is still unclear and will likely need to be interpreted within the context of a better understanding of patterns of cumulative exposure, environmental accumulation, and physiologic metabolism of these chemicals across the life span.

Several studies have been conducted to investigate the possible modes of action of PFOS. Induction of peroxisome proliferation and associated peroxisomal enzymes [19], activation of nuclear receptors [9], interference in lipid metabolism and decreases in serum cholesterol [10, 12, 13], and alterations in thyroid hormone homeostasis [20, 21] have all been investigated as possible modes of action. However, at the moment, the mechanisms of action related to the toxicity of PFOS are still not clearly understood.

T1DM is an autoimmune disease driven by the activation of T lymphocytes, mainly CD8^+^, against pancreatic *β*-cells. The lymphocytes count in our patients with diabetes was significantly lower than in control, with no apparent change in the number of circulating WBC. The worldwide increasing incidence of T1DM in childhood remains unexplained. Environmental chemicals that can act as endocrine disruptors may affect the development and function of the immune system in ways that could promote autoimmunity and thereby contribute to the development of T1DM [22].

The specific role of PPARs in PFCs-immunotoxicity is still a matter of debate, and it is unclear whether or not there is a direct effect on immune cells. In mice, PFOS and PFOA probably exert an influence on the immune system, activating innate immunity and suppressing adaptive immune responses but the cause and significance of this activation of immune system by PFCs remain to be determined [23]. On the other hand, various leukocyte populations express PPARs and therefore a role in immune response of lymphocytes is suggested for this transcription factor [24]. PFCs directly affected immune cell activation and reduced cytokine production (both pro- and anti-inflammatory) through different mechanisms as demonstrated by Corsini et al. [25]. The exact role of PPARs in PFCs-immunotoxicity is complex with some effects resulting from a PPAR-mediated mechanism, while other effects result from PPAR-independent mechanism.

Despite the fact that we observed that T1DM patients had significantly higher levels of TC compared to controls we only found a negative correlation between serum PFOS and TG. Considering the whole study population, multivariate linear regression analysis allowed us to identify lipid profile as main predictor factor of PFOS levels. Probably PFCs bind to the PPARs interfering with lipid metabolism because of their ability to act as PPAR agonists, as previously suggested from animal studies [19]. Few is known about how these chemicals interfere with the human biological mechanisms, but epidemiologic studies have consistently shown that PFCs, mainly PFOA, were positively associated with lipid profile [12, 26–28] while others have not had significant evidence to support the association with cholesterol outcomes with PFOA and PFOS [29]. Frisbee et al. [13] reported for the first time in children and adolescents that increasing PFOA and PFOS quintiles were positively associated with an increased risk of abnormal TC and LDL but not TG.

A limitation of our study is the small sample size. However, if associations reported in this paper are etiologic, exposure prevention would become important to reduce onset of T1DM and the long-term health consequences. Moreover, studying potential health consequences of an environmental exposure in children and adolescents may provide greater insight because these groups likely have fewer factors confounding underlying associations compared with adults. Finally, given possible differences in physiologic processes owing to developmental changes in children and adolescents, toxic effects may be different compared with those observed in adults.

In conclusion, our results suggest that high serum levels of PFOS may be considered a biomarker of susceptibility to develop TIDM.

## Figures and Tables

**Table 1:** Anthropometric characteristics of study population.

Groups/auxological data	T1DM (*n* 25)	Controls (*n* 19)	*p* value
Age (years)			0.001
Mean ± SD	8.04 ± 2.90	10.7 ± 2.77	
Median	8.28	11.2	
GM	7.46	10.1	
Height (cm)			0.970
Mean ± SD	127.7 ± 17.4	128.0 ± 12.6	
Median	132.0	131.9	
GM	126.6	127.4	
Weight (kg)			0.546
Mean ± SD	28.3 ± 9.92	29.5 ± 8.17	
Median	28.0	29.2	
GM	26.7	28.4	
BMI (kg/m^2^)			0.356
Mean ± SD	16.9 ± 2.27	17.7 ± 2.78	
Median	15.9	17.1	
GM	16.8	17.5	
*z*-BMI			0.273
Mean ± SD	0.21 ± 0.94	0.01 ± 1.04	
Median	0.00	−0.15	
GM	—	—	

BMI: body mass index; *z*-BMI: *z*-score body mass index; GM: geometric mean; SD: standard deviation; SDS: standard deviation score; T1DM: type 1 diabetes.

**Table 2:** Serum chemistry biomarkers and hematologic variables in study population.

Group/biochemical data	T1DM (*n* 25)	Controls (*n* 19)	*p* value
TSH (*μ*IU/mL)			0.375
Mean ± SD	2.39 ± 0.98	2.03 ± 0.50	
Median	2.24	1.98	
GM	2.22	1.98	
AST (U/L)			0.005
Mean ± SD	23.2 ± 3.09	35.3 ± 23.8	
Median	23.0	27.5	
GM	22.9	31.0	
ALT (U/L)			0.110
Mean ± SD	18.1 ± 4.90	25.0 ± 15.1	
Median	18.0	24.5	
GM	17.3	21.4	
TC (mg/dL)			0.015
Mean ± SD	183.7 ± 27.0	170.1 ± 15.1	
Median	188.0	170.5	
GM	181.6	169.5	
HDL-C (mg/dL)			0.622
Mean ± SD	62.7 ± 13.4	62.3 ± 8.05	
Median	62.0	61.5	
GM	61.1	61.9	
TG (mg/dL)			0667
Mean ± SD	65.0 ± 22.1	57.0 ± 9.85	
Median	60.0	56.0	
GM	61.9	56.3	
LDL-C (mg/dL)			0.597
Mean ± SD	101.1 ± 29.8	102.0 ± 12.1	
Median	101.0	102.0	
GM	96.8	101.3	
Urea (mg/dL)			0.112
Mean ± SD	32.1 ± 7.34	28.3 ± 4.88	
Median	34.0	28.0	
GM	31.3	28.0	
Creatinine (mg/dL)			0.631
Mean ± SD	0.60 ± 0.16	0.57 ± 0.11	
Median	0.59	0.56	
GM	0.59	0.57	
WBC (migl/*μ*L)			0.453
Mean ± SD	6.59 ± 1.72	7.08 ± 1.89	
Median	6.59	6.85	
GM	6.39	6.87	
Lymphocytes (migl/*μ*L)			0.008
Mean ± SD	2.28 ± 0.60	2.95 ± 0.85	
Median	2.12	3.00	
GM	2.21	2.83	

ALT: alanine aminotransferase; AST: aspartate aminotransferase; GM: geometric mean; HDL: high-density lipoprotein cholesterol; LDL: low-density lipoprotein cholesterol; SD: standard deviation; TC: total cholesterol; TG: triglycerides; T1DM: type 1 diabetes; TSH: thyroid-stimulating hormone.

**Table 3:** Serum concentration of PFOA and PFOS (ng/mL) in study population.

	*N*	Mean ± SD	Median	GM	*p* value	Min	Max	25th	75th	95% CI
PFOA (ng/mL)					0.160					
T1DM	25	0.53 ± 0.10	0.49	0.52		0.46	0.83	0.48	0.55	0.07–0.13
Control	19	0.50 ± 0.06	0.48	0.50		0.45	0.67	0.46	0.50	0.05–0.10
PFOS (ng/mL)					<0.001					
T1DM	25	1.53 ± 1.51	0.95	1.09		0.48	6.68	0.50	1.84	1.18–2.10
Control	19	0.56 ± 0.16	0.49	0.54		0.47	0.93	0.48	0.50	0.12–0.23

GM: geometric mean; PFOA: perfluorooctanoic acid; PFOS: perfluorooctane sulfonate; SD: standard deviation; T1DM: type 1 diabetes.

**Table 4:** Results of multivariate linear regression analysis on PFOS and PFOA levels in serum.

	PFOS			PFOA		
	All population (*n* 44)	T1DM (*n* 25)	Controls (*n* 19)	All population (*n* 44)	T1DM (*n* 25)	Controls (*n* 19)
SE	1.21	1.35	0.15	0.08	0.09	0.06
*R* (*R* ^2^)	0.43 (0.18)	0.63 (0.40)	0.64 (0.41)	0.35 (0.12)	0.50 (0.26)	0.63 (0.39)
*p* value	0.269	0.117	0.332	0.549	0.435	0.380

Intercept						
Coeff.	1.01	2.84	0.41	0.71	0.85	0.42
SE	1.97	2.58	0.50	0.14	0.18	0.21
*p* value	0.613	0.285	0.430	<0.001	<0.001	0.069

Age						
Coeff.	0.13	0.43	0.17	−0.24	−0.07	−0.20
SE	0.16	0.19	0.25	0.16	0.21	0.25
*p* value	0.404	0.035	0.523	0.143	0.751	0.449

BMI *z*-score						
Coeff.	−0.11	−0.21	−0.01	0.02	−0.23	0.43
SE	0.17	0.20	0.29	0.17	0.22	0.29
* p* value	0.507	0.306	0.968	0.889	0.322	0.172

TC						
Coeff.	0.82	0.02	0.44	−0.20	−0.86	0.12
SE	0.38	0.60	0.70	0.39	0.67	0.72
* p* value	0.040	0.972	0.544	0.614	0.219	0.864

HDL-C						
Coeff.	−0.34	−0.14	−0.70	−0.10	0.09	−0.20
SE	0.24	0.38	0.39	0.25	0.42	0.39
* p* value	0.172	0.719	0.097	0.692	0.834	0.618

TG						
Coeff.	−0.44	−0.47	0.47	−0.14	−0.07	0.09
SE	0.19	0.27	0.29	0.20	0.30	0.30
* p* value	0.029	0.099	0.141	0.496	0.804	0.753

LDL-C						
Coeff.	−0.74	−0.04	−0.24	0.22	0.75	0.19
SE	0.37	0.61	0.62	0.38	0.68	0.63
* p* value	0.054	0.946	0.706	0.559	0.288	0.765

*z*-BMI: *z*-score body mass index; HDL: high-density lipoprotein cholesterol; LDL: low-density lipoprotein cholesterol; PFOA: perfluorooctanoic acid; PFOS: perfluorooctane sulfonate; SE: standard error; TC: total cholesterol; TG: triglycerides; T1DM: type 1 diabetes.
